# Artificial Intelligence in Health Care: Bibliometric Analysis

**DOI:** 10.2196/18228

**Published:** 2020-07-29

**Authors:** Yuqi Guo, Zhichao Hao, Shichong Zhao, Jiaqi Gong, Fan Yang

**Affiliations:** 1 School of Social Work University of North Carolina at Charlotte Charlotte, NC United States; 2 School of Social Work The University of Alabama Tuscaloosa, AL United States; 3 Social Welfare Program School of Public Administration Dongbei University of Finance and Economics Dalian China; 4 Department of Information Systems University of Maryland Baltimore, MD United States

**Keywords:** health care, artificial intelligence, bibliometric analysis, telehealth, neural networks, machine learning

## Abstract

**Background:**

As a critical driving power to promote health care, the health care–related artificial intelligence (AI) literature is growing rapidly.

**Objective:**

The purpose of this analysis is to provide a dynamic and longitudinal bibliometric analysis of health care–related AI publications.

**Methods:**

The Web of Science (Clarivate PLC) was searched to retrieve all existing and highly cited AI-related health care research papers published in English up to December 2019. Based on bibliometric indicators, a search strategy was developed to screen the title for eligibility, using the abstract and full text where needed. The growth rate of publications, characteristics of research activities, publication patterns, and research hotspot tendencies were computed using the HistCite software.

**Results:**

The search identified 5235 hits, of which 1473 publications were included in the analyses. Publication output increased an average of 17.02% per year since 1995, but the growth rate of research papers significantly increased to 45.15% from 2014 to 2019. The major health problems studied in AI research are cancer, depression, Alzheimer disease, heart failure, and diabetes. Artificial neural networks, support vector machines, and convolutional neural networks have the highest impact on health care. Nucleosides, convolutional neural networks, and tumor markers have remained research hotspots through 2019.

**Conclusions:**

This analysis provides a comprehensive overview of the AI-related research conducted in the field of health care, which helps researchers, policy makers, and practitioners better understand the development of health care–related AI research and possible practice implications. Future AI research should be dedicated to filling in the gaps between AI health care research and clinical applications.

## Introduction

From its birth in the 1950s to present, artificial intelligence (AI) and its application in modern health care have boomed with the advancement of science and technology [1-3]. As a critical driving power that promotes the coming and development of industry 4.0, AI has become an indispensable component of the advancement and innovation of health care and medical diagnosis. Medical AI technologies provide algorithms and programs that analyze symbolic models of diseases and their relationships to patient signs and symptoms [4-6].

In the field of health care, the implementation of AI technologies fosters the prediction, diagnosis, and treatment of diseases, which benefits both patients and health care providers [7]. The promise of improving diagnostic accuracy is one of AI's most exciting health care applications. AI can effectively assist health care providers in diagnosing symptoms at a faster rate than most medical professionals [8]. AI can mimic the predictive power of human doctors to improve the accuracy of diagnosis by horizontally and vertically assessing the electronic health records of patients in a short period of time [9]. A meta-analysis reported that the diagnostic sensitivity of AI was higher than that of dermoscopy (91% vs 88%) [10]. Additionally, AI can help patients keep track of complex symptoms, improve patients’ quality of life, and increase medication adherence [11]. For example, a randomized clinical trial study indicated that an AI platform successfully increased medication adherence in stroke patients on anticoagulation therapy by 50% [12].

The widespread application of AI in health care advances the processing of related research. The body of relevant literature grows rapidly. As a result, health care–related AI studies are thriving in recent health care literature. Although health care–related AI research has gained popularity, only a few bibliometric analyses focus on AI applications in specific types of health problems, such as depression [13]. A bibliometric analysis of general health care–related AI studies can depict a map that helps researchers better understand the development of health care–related AI research and the direction of patterns and trends in the future. Keeping abreast of the fast-growing body of health care–related AI studies helps practitioners and policy makers to seize the opportunities of applying AI interventions to promote the well-being of patients and their caregivers.

Bibliometrics is a measurable informatic method that analyzes the emerging trends and the knowledge structure within a certain field to obtain quantifiable, reproducible, and objective data [11]. Bibliometric analysis provides researchers and related stakeholders the opportunity to gain an informative understanding of the field of study and promotes interdisciplinary collaboration [14]. This study aims to provide a holistic view of health care–related AI research and the directions of future work to benefit patients and health care providers. Through an extensive and global review of literature on AI in health care, the purpose of this analysis is to examine the AI research focused on promoting health care. This analysis is an indispensable resource for researchers to have as an overview of the AI field in health care, which will help them to develop health care–related AI studies. This analysis is also an essential resource for someone who is less familiar with this field but is interested in AI applications in health care.

## Methods

### Search Strategy

Based on bibliometric indicators, we developed a search strategy in an iterative manner, starting from search terms used in the literature already known to us. This bibliometric analysis provided critical insights into the current state of health care–related AI research up to September 2019. The Web of Science (WoS) (Clarivate PLC) Core Collection was used to search all existing and highly cited AI publications. We opted for using the Science Citation Index (SCI) and the Social Science Citation (SSCI) databases in Web of Science, and then we conducted the temporal and spatial analysis, analysis of word co-occurrence, coauthorship analysis, and cocountry analysis.

Search keywords related to (1) AI technologies and (2) health care and medicine were identified from a preliminary literature review and consultation with a librarian. We entered the retrieval search string by combing keywords with Boolean operators: TI=((artificial intelligence) OR TI=(“data learning”) OR TI=(“machine learning”) OR TI=(“expert systems”) OR TI=(“fuzzy logic”) OR TI=(“computer vision”) OR TI=(“automatic programming”) OR TI=(“speech understanding”) OR TI=(“autonomous robots”) OR TI=(“intelligent tutoring”) OR TI=(“intelligent agents”) OR TI=(“neural network”) OR TI=(“voice recognition”) OR TI=(“text mining”) OR TI=(“electronic health record”)) AND (TS=(health) OR TS=(healthcare) OR TS=(medicine) OR TS=(mental health) OR TS=(behavior health)), Indexes=SCI-EXPANDED, SSCI Timespan=All years. The final search was conducted on September 09, 2019, in the WoS. A total of 5235 papers that were registered between January 1995 and September 2019 in the SCI Expanded and the SSCI Index databases from the Web of Science.

### Screening Strategy

In this analysis, all journal papers about AI in health care were included for screening. The papers for analysis were restricted to those that (1) were written in the English language, (2) focused on promoting health or health care, and (3) involved AI technologies. As AI technology is a leading-edge and rapid update research area, papers published in peer-reviewed journals, conference proceedings, and early access articles were included. Book chapters and books were excluded from this bibliometric analysis.

**Figure 1:**
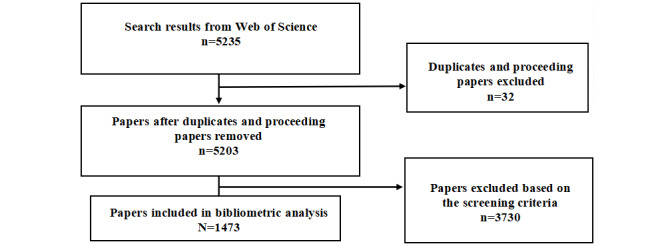
Flowchart detailing the paper collection and screening process.

The coauthors received training of bibliometric analysis screening by watching the video of Müller's study screening guide [15]. The screening procedure was conducted based on the screening guide [15]. Two coauthors (YG and ZH) conducted a pilot screening with the same set of 50 papers. During the pilot screening process, papers from the preliminary searches were categorized into include, exclude, or unsure. Papers marked as unsure were screened by 3 of the authors (YG, ZH, and FY) and discussed until consensus was reached in team meetings. The practical inclusion and exclusion criteria were determined. After that, 2 coauthors (ZH and YG) each received a unique set of papers for title and abstract screening. Different from a systematic review, a bibliometric analysis only requires screening of the abstract and full text when it is necessary. First, 32 inapplicable and duplicate papers were removed. According to the screening criteria, 3730 papers were excluded either because they did not focus on promoting health care or did not involve AI technologies. Finally, 1473 papers were included for bibliometric analysis (Figure 1).

### Bibliometric Analysis

Bibliometrics is the quantitative study of literature and a measurable method used to identify the developmental trends within a certain field to obtain quantifiable, reproducible, and objective data. In this analysis, we computed the growth rate of publications, characteristics of research activities (topics and keywords), publication patterns (countries and journals), and research hotspot tendencies (citation bursts and timeline map).

#### Growth Rate of Publications

The growth rate of publications over time was computed by raising the rate of the number of publications in 2019 over the number of publications in 1995 to the power of 1/24, as shown below. The publication trends of the number of publications each year were also reported.

Growth rate = ((number of publications in the last year ÷ number of publications in the first year)^1/(last year - first year)^ - 1) × 100

#### Publication Patterns

Citation trends of the top 10 countries, top 10 journals, and top 10 research domains were analyzed for the publication patterns. Frequencies and percentages of publications in each journal and each country were computed based on the publication years. The countries were identified by the affiliations of the listed authors. This information was provided by WoS. The rank of research impact of each country and each journal was provided based on the citation rates.

#### Characteristics of Research Activities

The characteristics of research activities were analyzed according to the topics and the keywords of publications. Top topics, including health problems, AI technology, function, and population, were identified and described by frequency, percentage, and citation rate of keywords listed by the authors. The centrality of a keyword was a combination of statistic equations that measure the representativeness of selected words for the text content according to betweenness, closeness, degree, eigenvector, PageRank (Google LLC), eccentricity, coreness, clustering coefficient, and term frequency scores [16]. The centralities of keywords were computed using HistCite. .

#### Research Hotspot Tendencies

Citation bursts and a timeline map were developed using the HistCite software. The cluster view was generated based on publications between 1995 and 2019, and each cluster was labeled by the keywords used by the paper. The time slice was set as 1 year and the threshold interpolation citations, cocitations, and cocitation coefficient were set as 4, 1, and 20, respectively. A minimum tree calculation formula was adopted to tailor the network.

## Results

### Growth Rate of Publications

**Figure 2:**
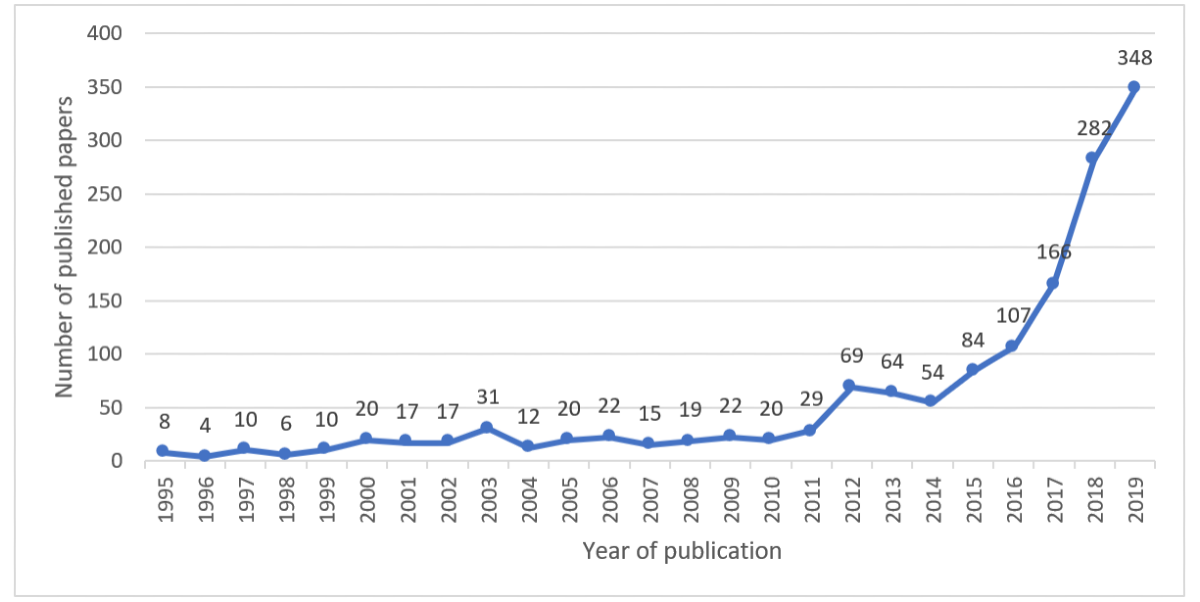
The distribution of the bibliographic records per year.

Figure 2 plots the annual trends of publications about AI in health care. From 1995 to 2019, the average growth rate of scientific research papers on health care–related AI research was 17.02%. The growth rate from 1995 to 2010 was 6.33%, the growth rate from 2011 to 2014 was 23.02%, and the growth rate from 2015 to 2019 was 42.67%. The number of publications increased steeply between 2014 and 2019, accounting for 70.67% (1041/1473) of all included papers.

### Publication Patterns

Overall, 95.59% (1408/1473) of the included papers were published from 10 countries, shown in Table 1. About 45.42% (669/1473) of the included studied were from the United States. China was the next leading country (183/1473, 12.42%), followed by England (113/1473, 7.67%). The papers were published in 715 different journals. As demonstrated in Table 2, *PLOS One* published the most papers (57/1473, 3.87%), followed by *Artificial Intelligence in Medicine* (24/1473, 1.63%), and *Expert Systems with Applications* (1.63%, 24/1473). WoS subject categories were employed to indicate the research domains of included publications, as seen in Table 3. The research domains of computer science (252/1473, 17.11%), engineering (192/1473, 13.03%), and medical informatics (169/1473, 11.47%) are the top research domains of health care–related AI research.

**Table 1:** The distribution of the bibliographic records by top 10 (by quantity) countries.

Countries	Ranking based on total output	Output^a^, n (%)	Ranking based on citations	Citations^b^, n (%)
United States	1	669 (47.79)	1	10,794 (51.11)
China	2	183 (13.07)	2	2568 (12.16)
England	3	113 (8.07)	3	1969 (9.32)
India	4	82 (5.86)	9	598 (2.83)
Italy	5	74 (5.29)	5	924 (4.37)
Germany	6	73 (5.21)	4	1462 (6.92)
Canada	7	63 (4.50)	6	823 (3.90)
Japan	8	49 (3.50)	6	823 (3.90)
Spain	9	48 (3.43)	8	724 (3.43)
Iran	10	46 (3.29)	10	438 (2.07)

^a^N=1400.

^b^N=21,123.

**Table 2:** The distribution of the bibliographic records by top 10 (by quantity) journals.

Journals	Ranking based on total output	Output^a^, n (%)	Ranking based on citations	Citations^b^, n (%)
*PLOS One*	1	57 (23.55)	5	395 (9.85)
*Artificial Intelligence in Medicine*	2	24 (9.92)	1	923 (23.01)
*Expert Systems with Applications*	3	24 (9.92)	3	566 (14.11)
*Scientific Reports*	4	24 (9.92)	10	71 (1.77)
*Journal of the American Medical Informatics Association*	5	22 (9.09)	4	426 (10.62)
*Journal of Medical Systems*	6	21 (8.68)	7	262 (6.53)
*Computer Methods and Programs in Biomedicine*	7	20 (8.26)	8	222 (5.53)
*Medical Physics*	8	19 (7.85)	2	689 (17.17)
*Journal of Biomedical Informatics*	9	16 (6.61)	6	319 (7.95)
*Computers in Biology and Medicine*	10	15 (6.20)	9	139 (3.46)

^a^N=242.

^b^N=4012.

**Table 3:** The distribution of the bibliographic records by top 10 (by quantity) research domains.

Research domains	Ranking based on total output	Output^a^, n (%)	Ranking based on citations	Citations^b^, n (%)
Computer science	1	252 (18.42)	1	15,706 (21.01)
Engineering	2	192 (14.04)	6	5468 (7.32)
Medical informatics	3	169 (12.35)	8	4893 (6.55)
Oncology	4	153 (11.18)	2	11,467 (15.34)
Radiology, nuclear medicine, and medical imaging	5	142 (10.38)	4	6989 (9.35)
Health care sciences services	6	132 (9.65)	5	6729 (9.00)
Science, technology, and other topics	7	99 (7.24)	7	5207 (6.97)
General internal medicine	8	85 (6.21)	10	2565 (3.43)
Mathematical and computational biology	9	78 (5.70)	3	10,894 (14.57)
Biochemistry and molecular biology	10	66 (4.82)	9	4831 (6.46)

^a^N=1368.

^b^N=74,749.

### Characteristics of Research Activities

Keywords are the core word extractions provided by researchers in the studies. Table 4 shows information about the frequency and centrality of keywords. The top 5 health problems are cancer, depression, Alzheimer disease, heart failure, and diabetes. The top 5 AI technologies are machine learning, artificial neural networks, deep learning neural networks, electronic health records, and support vector machines. The top 5 functions are case classification, diagnosis, prediction, risk estimate, and chronic condition management. The top 5 populations focused on in health care–related AI studies are children, adults, women, men, and elderly persons.

**Figure 3:**
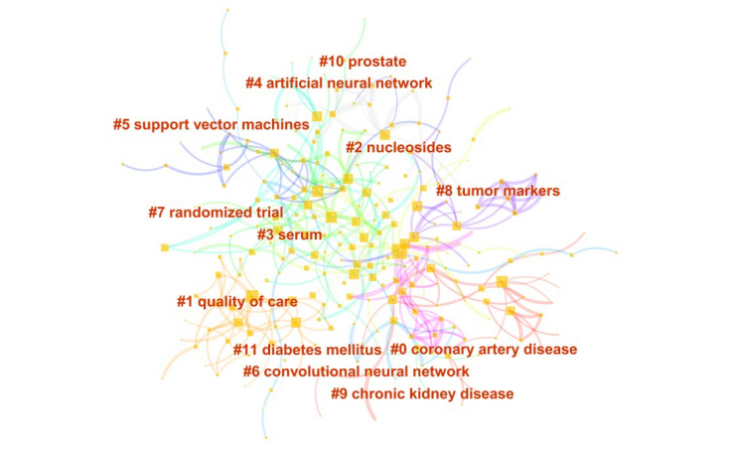
Cluster analysis of artificial intelligence health care publications.

HistCite intelligently classified the research topic into 12 clusters, labeled from 0 to 11 in Figure 3. The modularity (Q) was 0.423, which was higher than 0.3, indicating that the cluster results were significant. Cluster 0 is the largest cluster (coronary artery disease) and cluster 11 is the smallest one (diabetes mellitus). Each cluster was generated based on the number of keywords under one research domain, not the frequency of keywords.

**Table 4:** The top keywords of artificial intelligence health care publications.

Category		Frequency (as identified by title, keywords, or manuscript)	Centrality
**Health problem**			
	Cancer^a^	273	0.13
	Depression	16	0.02
	Alzheimer disease	7	0.00
	Heart failure	5	0.00
	Diabetes	3	0.00
**Technology**			
	Machine learning	288	0.09
	Artificial neural network	270	0.13
	Deep learning neural network	95	0.01
	Electronic health record	87	0.06
	Support vector machine	62	0.03
**Function**			
	Case classification	269	0.11
	Diagnosis	165	0.14
	Prediction	149	0.06
	Risk estimate	116	0.10
	Chronic condition management	71	0.02
**Population**			
	Children	25	0.01
	Adult	15	0.00
	Women	11	0.00
	Men	9	0.00
	Elderly persons	7	0.00

^a^Breast: n=124; carcinoma: n=46; prostate: n=45; lung: n=44; other: n=14.

### Research Hotspot Tendencies

**Figure 4:**
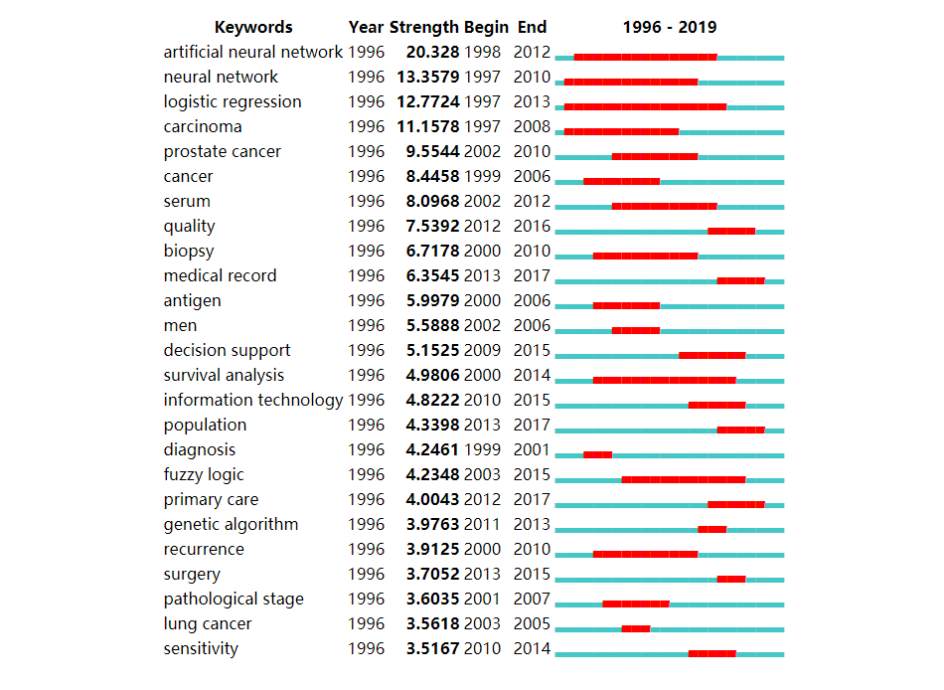
Top 15 keywords with the strongest citation bursts.

We presented the major milestones in the development of AI in health care by analyzing the list of keywords that had strong citation bursts between 1996 and 2019, as seen in Figure 4. The first milestone keywords in the studies were neural network, logistic regression, and carcinoma. The next milestone was the artificial neural network (highly cited in 1998-2002). The most recent milestone was survival analysis, highly cited until 2014.

We also generated a timeline visualization to depict clusters along a horizontal axis, seen in Multimedia Appendix 1. The clusters we analyzed in Figure 3 were vertically listed in descending order of their sizes on the right of Multimedia Appendix 1. Each cluster was analyzed on each horizontal timeline from left to right, with the year shown on top of the view. The colored curves represent cocitation links added in the year of the corresponding color. Large-sized nodes are of particular interest because they are either highly cited, have citation bursts, or both.

## Discussion

By using bibliometric data, this study analyzed the health care–related AI research through examining the growth rate of publications, characteristics of research activities, publication patterns, and research hotspot tendencies.

### Growth Rate of Publications

Since the first publication in 1995, health care–related AI research had a slow increase in the following 17 years. However, since 2012, the field has started to move forward at a fast pace. In the past 5 years, the growth rate of publication reached 45.1%, which is more than 4 times the publication growth rate from 1995 to 2014. The recent rapid growth is due to multiple reasons. The technological breakthroughs of AI in this time period contributed to the explosive growth of AI adoption in health care–related AI research [17]. From 2015 to 2018, the appearance of autonomous robots, voice recognition, neural networks, and machine learning provided unprecedented opportunities for the prediction, diagnosis, and treatment or management of diseases [18,19]. According to the exponential growth pattern of health care–related AI research, the publications in health care–related AI will continue to grow in the future. Applying the growth rate of the past 5 years to the next 5 years, we can estimate that the publication volume in health care–related AI research will double about every two years.

### Publication Patterns

The field of AI health care has attracted people from all around the world, but the high-income countries are the main force in health care–related AI research. The United States itself contributed about half of the research in the field of health care–related AI research. Compared with the rapid advancement of health care–related AI research, this research field in non–high-income countries is still meager. This observation of the small outputs in middle-income and low-income countries causes concern because many low-income countries have limited health care resources, but their public health issues are nonetheless increasing dramatically due to rapid globalization and urbanization [20]. Some developed countries have launched federal AI health care policies incorporating guidance on the development and regulation of AI in health care, such as the UK Code of Conduct for Data-Driven Health and Care Technology [21]. Consequently, about 85% of the research outputs were generated in developed countries, but 80% of the world population lives in developing countries. Multiple barriers, including funding, prioritization, research capacity, infrastructure, and language contribute to these disproportionate results [22]. From a global perspective, AI technologies are promising in terms of promoting health outcomes in low-income countries with limited health care resources.

Based on the output and citation counts, health care–related AI research is generally favored by large-scale journals related to health care. However, the sizes of the research effect are field-specific. The improvement and sophistication of the fields of computer science and engineering have paved the way for the development of AI. It is worth noting that the field of oncology received the second most citations. These results are consistent with the findings of the top keywords of included papers, implying that published AI papers on cancer care accumulated a high number of citations.

### Characteristics of Research Activities

AI is redefining and disrupting the way health care is being carried out across diverse levels, based on the results of the characteristics of AI publications. According to the top keywords in the identified categories, the top domains of disease in AI research are cancer, coronary artery disease, chronic kidney disease, prostate cancer, and diabetes mellitus*.* The diseases of the leading causes of death received the major attention of AI research. By 2030, chronic diseases will contribute 80% of the human deaths globally and result in severe global burden of disease [23-25]. Specifically, the cancer mortality rate has steadily risen by 6% during the past 10 years [26]. Therefore, researchers direct their hopes and efforts on early detection and condition management by using advanced technology [27,28]. AI is making its way into cancer treatment from diagnostic classification to tumor behavior prediction [29,30]. Furthermore, cardiovascular diseases are also highly related to behaviors, especially among older adults [31,32]. AI can exert a proactive role in predicting risk factors for cardiovascular diseases related to older adults’ behaviors in order to remarkably reduce the hospitalization rate, readmission rate, cost of care, and even mortality rate [33-35].

Extensive functions of AI have assisted modern health care by providing smart medical data analysis and developing accurate and efficient prediction for treatment [36]. Machine learning is the most commonly used AI technique in health care. Artificial neural networks are the next biggest research area related to health care. There are promising applications of these AI technologies to the development of interventions for other diseases in future research, such as mental health interventions, health education, and chronic disease management.

AI research is witnessing widespread adoption in the prediction, detection, diagnosis, classification, treatment, and survival prediction of diseases [30,37]. The most common application of AI technologies is reflected in the domains of medical classification and quality of care. AI is investing significantly in improving the quality of care in the health care system [38]. The potential of AI in reviewing medical images and analyzing large-scale data has led to significant improvement in the quality of care [39]. Additionally, AI-based risk prediction models can investigate the complex relationships between clinical data and disease treatment [40].

Regarding the research populations, researchers seem more interested in child populations. Due to the development of cognitive aids to support diagnosis, treatment, care coordination, surveillance and prevention, and health maintenance, improvement of AI in clinical pediatric health care is remarkable [41]. However, cancer and various chronic diseases are the main focus of the current health care–related AI publications. Considering that elderly populations are the primary sufferers of chronic diseases, future studies may shift their angle to further explore AI implications on health in older adult populations.

### Research Hotspot Tendencies

In the last three decades, the times and lengths of citation bursts of each health care–related AI research topic have varied. Certain keywords were extraordinary consistent for a long period of time, while some keywords only briefly surged in the field of AI health care. The period of 1997 to 2014 contained a concentrated outbreak of multiple health care–related AI study bursts (Figure 4). AI technology research involved more health care fields and produced more diverse keywords in recent years. It is possible that the more diverse keywords of health care–related AI research diluted the citation bursts.

According to the citation rates, artificial neural networks, support vector machines, and convolutional neural networks have the highest impact on health care. These techniques have been widely adopted by oncology research to predict disease and survival rates [42]. Historically, logistic analysis has been a widely used research method of health care–related AI. However, survival analysis has had a recent citation burst in this field.

The studies in these clusters focus on testing AI techniques and translating these techniques into practical settings [37,43]. The next step of health care–related AI research may transform from lab-based research to the development of clinically validated and safe regulated systems. The current health care–related AI research faces the challenges of deploying lab-based AI intervention into clinical practice [44]. These challenges include ethical issues brought up by AI application [45] and the quality and quantity of medical data and cases [46], which will decide the diagnosis and treatment ability of AI.

Health care–related AI research experienced several paradigm shifts during the past decades and continues to shift. As shown in the timeline overview (Multimedia Appendix 1), the sustainability of health care–related AI research clusters is influenced by the increasing availability of health care data and the rapid progression of AI techniques. The research of cancer, convolutional neural networks, and nucleosides sustained a hotspot over the past twenty years, whereas some clusters were relatively short-lived, such as chronic kidney disease, prostate cancer, and diabetes mellitus. Particularly, nucleosides, convolutional neural networks, and tumor markers have remained research hotspots through 2019. These research domains exert strong impacts on the field and these analysis results suggest that their influence will likely continue in the next few years.

### Limitations

Limitations of our work need to be acknowledged. First, although we are confident that a single database—WoS—is a large enough database to offer a wide variety of publications vital for our analyses, future studies will apply other databases, such as Scopus, to explore more potential papers. Second, the search keywords (eg, mental health, behavioral health, and health care) related to health care were quite general, which may not be able to identify AI-related studies in all aspects of health care. For example, AI-related studies of HIV might have been omitted. Third, some keywords, although they were ranked as top keywords, were uninformative by themselves (eg, risk, model, and system) and could not be analyzed. Fourth, we did not include gray literature (eg, books), and we did not include papers published in languages other than English. Therefore, we may have missed relevant studies conducted in different forms, languages, and countries. Future studies can broaden the search scope to explore more relevant research to enrich the literature.

### Conclusions

We aimed to provide a bird’s-eye view of the entirety of the health care–related AI research. This analysis provides a comprehensive overview of the AI-related research conducted in the field of health care. With multiple searching and screening rounds, 21 finalized search terms, and a 25-year timespan from 1995 to 2019, we are confident that we have identified inclusive health care–related AI studies.

This analysis also depicted research trends of AI-related health research: (1) the growth rate of health care–related AI publications has grown rapidly in the past decade and the rate showed a trend of continuous growth; (2) high-income countries are the main force of health care–related AI research; (3) most AI research was focused on chronic diseases, particularly on cancer; (4) machine learning and neural networks are the most commonly used AI techniques in classification, diagnosis, and prediction; and (5) the research domains of nucleosides, convolutional neural networks, and tumor markers are currently research hotspots of health care–related AI research. AI research on health care is accelerating rapidly, with potential applications being demonstrated across various domains of medicine. However, there are currently limited examples of such techniques being successfully deployed into clinical practice. Future AI research should be dedicated to filling the gap between health care–related AI research and clinical applications.

## Appendix

Multimedia Appendix 1 The keywords timeline view of artificial intelligence health care publications.

## Abbreviations

AI: artificial intelligence
SCI: Science Citation Index
SSCI: Social Science Citation
WoS: Web of Science
