# Supplementary figures and images for: Artificial Intelligence in Health Care: Bibliometric Analysis
Source: J Med Internet Res. 2020 Jul 29;22(7):e18228. doi: 10.2196/18228 (PMC7424481; doi:10.2196/18228)

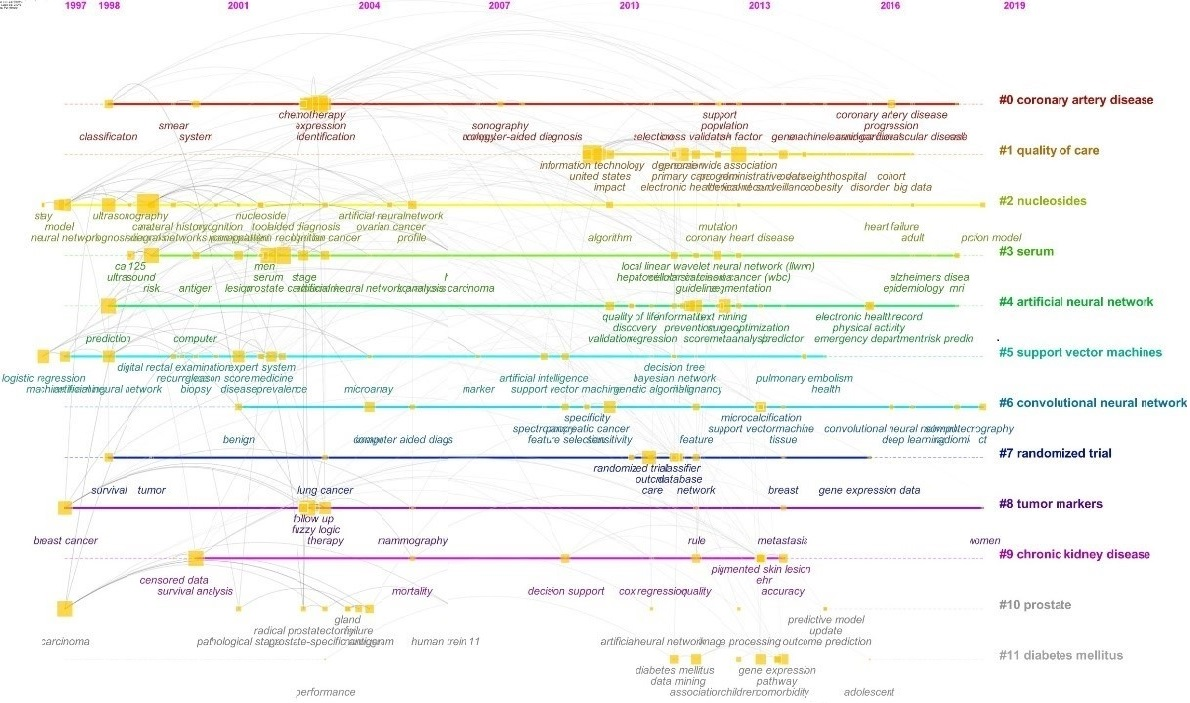

Supplement: Multimedia Appendix 1 [file jmir_v22i7e18228_app1.png]
